# Serum Calprotectin Level as an Inflammatory Marker in Newly Diagnosed Hypertensive Patients

**DOI:** 10.1155/2022/6912502

**Published:** 2022-01-21

**Authors:** Nergiz Bayrakci, Gülsüm Ozkan, Sonat Pinar Kara, Ahsen Yilmaz, Savas Guzel

**Affiliations:** ^1^Tekirdag Namik Kemal University, School of Medicine, Department of Nephrology, Tekirdag, Turkey; ^2^Tekirdag Namik Kemal University, School of Medicine, Department of Internal Medicine, Tekirdag, Turkey; ^3^Tekirdag Namik Kemal University, School of Medicine, Department of Biochemistry, Tekirdag, Turkey

## Abstract

**Background:**

Hypertension is one of the leading causes of cardiovascular mortality. Although the pathogenetic process involved is not yet fully understood, the disease involves endothelial damage and inflammation. Calprotectin is an inflammatory marker that rises in parallel with disease activity in conditions such as systemic inflammatory diseases, infection, and atherosclerosis. The purpose of this study was to evaluate inflammation through serum calprotectin levels in newly diagnosed primary hypertension patients.

**Methods:**

Forty-nine newly diagnosed hypertensive patients and 38 healthy adults were included in the study. Patients' office blood pressure values, biochemical findings, and demographic characteristics were recorded. Serum calprotectin levels were measured using ELISA. Parameters affecting serum calprotectin levels and determinants of hypertension were evaluated.

**Results:**

Serum calprotectin levels were 242.8 (72.4–524) ng/mL in the control group and 112.6 (67.4–389.8) ng/mL in the hypertensive patient group, the difference being statistically significant (*p*=0.001). There was no correlation between serum calprotectin levels and other parameters (blood pressure values, age, gender, serum creatinine, uric acid, and calcium levels) in the hypertensive group. A lower serum calprotectin level was found to be independently related to hypertension (*β* = −0.009, *p*=0.005). Serum calprotectin at a cutoff level of 128.6 ng/mL differentiated hypertensives from healthy controls with a sensitivity of 69.4% and specificity of 68.4% (AUC = 0.767).

**Conclusions:**

The results of this study were the opposite of our hypothesis that a higher calprotectin level may reflect subclinical endothelial damage in newly diagnosed hypertensive patients. Further comparative studies involving patients at different stages of hypertension may contribute to clarifying the relationship between calprotectin and hypertension. We conclude that molecular studies seem essential for understanding the place of calprotectin in hypertension-associated inflammation, a complex process.

## 1. Introduction

Hypertension is one of the main modifiable risk factors in terms of cardiovascular diseases [1]. The search for inflammatory markers involved in the pathogenesis of primary hypertension and associated end organ damage is still ongoing. Although this has not yet been fully elucidated, several previous studies have shown the presence of inflammation and oxidative stress-mediated endothelial damage in hypertension [2]. Inflammatory molecules such as angiotensin II (Ang II), tumor necrosis factor alpha (TNF-*α*), interleukin (IL)-1, IL-2, and IL-6, and monocyte chemoattractant protein-1 (MCP-1) have been associated with hypertensive cardiovascular injury, although it is unclear which of these and similar markers in fact trigger inflammation in the early stage of injury [3, 4]. It is anticipated that potential markers will contribute to both the noninvasive identification of end organ damage and to the development of treatments aimed at reducing hypertension-related morbidity and mortality [5, 6].

Calprotectin is a multipotent calcium-binding protein weighing 36.5 kDa, exhibiting immunomodulatory, antiproliferative, and proinflammatory efficacy, and consisting of a S100A9 and S100A8 molecule complex from the S100 family, found in monocyte, macrophage, and dendritic cells and particularly in neutrophil cytoplasm [7, 8]. Dramatic increases in calprotectin levels occur in body fluids in conditions progressing with acute endothelial damage, such as acute coronary ischemia, preeclampsia, and, particularly, Th1 lymphocyte-mediated immune response, and in acute exacerbations of chronic inflammatory diseases such as inflammatory arthritis and inflammatory bowel diseases [9–12]. Calprotectin levels have also been reported to rise in chronic diseases such as cystic fibrosis, connective tissue diseases, cancer, and heart failure [13–15]. Some studies have investigated calprotectin levels in primary hypertension patients, and the relationship between calprotectin and high blood pressure and end organ damage remains unclear [16–18].

Although calprotectin is generally described as an acute inflammatory marker, in the light of the endothelial damage mechanisms in the pathogenesis of hypertension, it has also been proposed that serum calprotectin levels may be a marker of the inflammatory process in hypertension. The purpose of this study was to evaluate serum calprotectin levels as an early inflammatory marker in newly diagnosed primary hypertension patients.

## 2. Methods

### 2.1. Patient Selection and Data Collection

Eighty-seven adults, consisting of 49 newly diagnosed hypertensive patients presenting to a tertiary nephrology outpatient clinic between December 2019 and April 2020 and 38 healthy controls presenting to the general internal medicine outpatient clinic, were included in this cross-sectional, observational study. The exclusion criteria were age under 18 and presence of chronic drug use, active infection, acute kidney injury, and any chronic systemic disease including cardiovascular disease, chronic kidney disease, or malignancy. The study was conducted in accordance with the Declaration of Helsinki and was approved by the Local Ethics Research Committee (research protocol no. 2019.213.11.10; approval dated 26.11.2019). All subjects provided written informed consent prior to participation in the study.

Peripheral blood samples for the measurement of biochemical parameters and calprotectin were collected in the morning following at least 8 h fasting. Serum specimens obtained by centrifuging the blood samples at 2500 × g for 10 min were stored at −80°C. Serum calprotectin levels were measured using a Bioassay Technology Laboratory (Shanghai Korain Biotech Co. Ltd., Shanghai, China) commercial ELISA kit (catalog no. E4010Hu; sensitivity: 1.67 ng/ml; intra-assay coefficient of variation (CV) <8%; interassay CV <10%).

### 2.2. The Assessment of Blood Pressure

Office blood pressure monitoring was applied for the diagnosis of hypertension, and measurements were interpreted according to the ESC/ESH 2018 guideline. Office BP was measured using a UA-651SL monitor (A&D Company, 1-243 Asahi, Kitamoto-shi, Saitama-ken, 364-8585, Japan), a validated device. Before the procedure, all patients were asked to rest for at least 5 min in a relaxed position in a quiet room at a comfortable temperature. They were also asked whether they had consumed any caffeine, alcohol, or cigarettes in the previous 30–60 min. BP was measured by a physician from both arms using a cuff of a suitable size for the patient's forearm, with the forearm held at the level of the heart, with the back and the forearm supported, and with the patient sitting upright. We were careful to ensure that patients did not cross their legs or speak during the procedure. Once BP had been measured from both arms, subsequent BP measurements were carried out using the arm eliciting the highest value. BP was measured twice at 1 min intervals. The mean value of the two measurements was recorded as office BP. Hypertension is defined as office systolic blood pressure (SBP) values >_140 mmHg and/or diastolic blood pressure (DBP) values >_90 mmHg. Diagnosis was confirmed through ambulatory blood pressure monitoring (ABPM) when office measurement was consistent with hypertension. A Mobil-O-Graph NG 24 h ABPM Classic (IEM GmbH, Stolberg, Germany) device was used for 24 h ABPM measurement. Patients recorded the times they spent sleeping, waking, and eating, together with daily activities performed. Patients were also asked to ensure that the arm was kept immobile during BP measurement. Daytime BP measurement was performed at 15 min intervals and nighttime measurement at 30 min intervals. Subjects with at least 70% measurement in 24 h ABPM records were included in the analysis. The diagnostic threshold for hypertension was ≥130/80 mmHg over 24 h, ≥135/85 mmHg for the daytime average, and ≥120/70 mmHg for the nighttime average [19]. If indicated, patients were screened for secondary hypertension. Since ABPM was not performed in the normotensive group, office blood pressure measurements of both groups were included in the analyses.

### 2.3. Statistical Analysis

Data were analyzed on Statistical Package of Social Science (SPSS) 25.0 for Windows software (SPSS Inc., Chicago, IL, USA). Variable distributions were assessed using the Kolmogorov–Smirnov normality test. Comparison of continuous variables between two groups was performed using the independent-sample *T*-test or Mann–Whitney *U* test based on the normality test results. Categorical variables were compared using the chi-square test. Correlation between two continuous variables was assessed using Pearson's or Spearman's correlation coefficients. Linear regression was used to evaluate the parameters affecting serum calprotectin levels. Logistic regression analysis was applied to determine risk factors for hypertension. Multivariate analysis was adjusted for confounders. Data were expressed as “mean ± SD” or “median (min–max).” A receiver operating characteristic (ROC) curve was constructed to determine the optimal calprotectin level cutoff value for differentiating hypertensives and healthy controls. *p* values less than 0.05 were regarded as statistically significant.

## 3. Results

### 3.1. Baseline Characteristics

Forty-nine patients with primary hypertension (age: 44.98 ± 11.12) and 38 healthy individuals (age: 44.66 ± 7.82) were included in the study. The number of women was significantly higher in the control group compared to the hypertensive group (*p* < 0.001). Serum creatinine levels were significantly higher and glomerular filtration rate (GFR) values were significantly lower in the hypertensive group compared to the control group (*p*=0.001 and *p*=0.04, respectively). Serum uric acid, C-reactive protein (CRP), and calcium levels were also significantly higher in the hypertensive group (*p*=0.001, *p*=0.003, and *p*=0.023, respectively). Serum calprotectin levels were 242.8 (72.4–524) ng/mL in the control group and 112.6 (67.35–389.77) ng/mL in the hypertensive group, the difference being statistically significant (*p*=0.001). Patients' demographic characteristics and laboratory values are shown in Table 1.

### 3.2. Relationship between Calprotectin Levels and Other Parameters

When correlation analysis was performed across the entire study group, mean systolic blood pressure (SBP), diastolic blood pressure (DBP), and serum uric acid and calcium values were negatively correlated with serum calprotectin levels (*r* = −0.411, *p*=0.001; *r* = −0.377, *p*=0.001; *r* = −0.370, *p*=0.001; and *r* = −0.293, *p*=0.009, respectively). However, when the analysis was performed separately for the patient and control groups, no correlation was found between the calprotectin level and other parameters within the groups (Table 2). Despite the different gender distributions in the control and patient groups, no gender difference was observed in terms of serum calprotectin levels (*p*=0.263).

### 3.3. Parameters Affecting Serum Calprotectin Levels

Univariate and multivariate linear regression analyses, including biochemical and clinical parameters, were performed across the entire study group. SBP and serum uric acid levels emerged as independent parameters affecting serum calprotectin levels (*ß* = −0.54, *p*=0.009 and *ß* = −0.30, *p*=0.024, respectively), while no association was observed with DBP, serum creatinine, or serum calcium levels (Table 3).

### 3.4. Determinants of Hypertension

**Figure 1:**
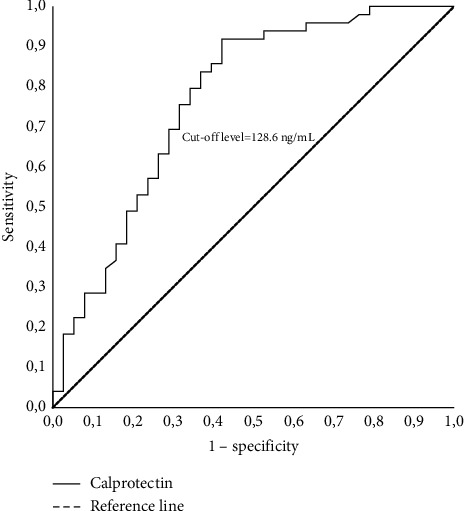
Receiver operating characteristic (ROC) curve for serum calprotectin for indicating hypertension (AUC = 0.767; *p* < 0.001; CI = 0.664–0.871).

Univariate and multivariate logistic regression analyses, including biochemical and clinical parameters, were performed across the entire study group. Serum calprotectin levels and eGFR were found to be independently associated with hypertension (*ß* = −0.01, *p*=0.005 and *ß* = -0.07, *p*=0.008, respectively), while no association was observed with gender, age, serum calcium, or uric acid levels (Table 4). Serum calprotectin levels lower than 128.6 ng/mL exhibited a sensitivity of 69.4% and specificity of 68.4% for hypertension (AUC = 0.767) (Figure 1).

## 4. Discussion

To the best of our knowledge, this is the first study investigating serum calprotectin levels in newly diagnosed hypertensive patients. Serum calprotectin levels were significantly higher in the control group than in the hypertensive group. This finding is inconsistent with the previous studies evaluating calprotectin in inflammation [9, 17, 20–22]. Serum calprotectin levels were independently associated with hypertension and were negatively affected by serum uric acid levels and SBP in the entire patient group. Although not compatible with the previous studies, we think that these findings, which seem to be consistent with one another, cannot be coincidental and therefore require interpretation. Serum calprotectin levels are reported to increase during inflammatory processes, more markedly in acute inflammation. Increased calprotectin secretion has been shown to occur as a result of the interaction between endothelial cells and cells involved in the inflammatory process. Several studies investigating inflammation and pathogenetic processes in hypertension have reported higher serum CRP, uric acid, and calcium levels [23–28]. Among these parameters, serum uric acid and calcium levels were found to exert a negative effect on serum calprotectin levels. We therefore concluded that low calprotectin levels are probably associated with the inflammatory process in hypertensive individuals. The first possibility in that context involves a sudden increase in neutrophil-derived calprotectin levels in hypertensive individuals, first in tissue and subsequently in serum, followed by a rapid decrease to normal limits. This may be due to the short neutrophil half-life. In a previous study investigating the early-stage inflammatory process in angiotensin II (Ang II)-induced hypertensive cardiac injury, an increase in 287 inflammatory genes was determined among the subjects, one of which was neutrophil-derived calprotectin. That study also reported that calprotectin led to receptor for advanced glycation end products (RAGE)-mediated damage in cardiac fibroblasts, that calprotectin-associated neutrophil levels rose suddenly on the first day following Ang II infusion, and that serum and tissue levels decreased gradually on the third and seventh days. The authors attributed this sudden fall to the short neutrophil half-life [29]. Another factor requiring consideration is whether the method used to determine calprotectin serum levels is appropriate to the study group. From that perspective, more careful examination of the molecular characteristics of calprotectin may assist with the maturation of this hypothesis. Calprotectin exists as a stable noncovalent heterodimer of S100A8/S100A9, which converts rapidly into a heterotetrameric form when calcium levels increase approximately 100-fold in the neutrophilic cytosol as a result of neutrophil stimulation [30–32]. The calprotectin molecule leaving the neutrophil has a heterotetrameric structure. It has been suggested that modification of the molecular structure of calprotectin in the extracellular environment is associated with neutrophil-derived oxidative damage and that oxidized calprotectin molecule is more sensitive to proteolysis. Low serum calprotectin levels may be detected as the molecule may have been rapidly broken down in the inflammation zone or during test specimen storage [32]. Similar to our results, a previous study investigating the relationship between the lipid profile and serum calprotectin level in patients with axial spondyloarthropathy reported higher calprotectin levels in healthy subjects. One of those authors' suggestions concerning this unexpected finding involved local accumulation of calprotectin in synovial fluid based on its small size [33]. It is possible that, for the reasons described above, the serum calprotectin levels in our hypertensive group may be insufficient to reflect the existing subclinical inflammation.

A negative correlation was determined between serum calprotectin and uric acid levels, with calprotectin levels being affected by uric acid levels. Uric acid has been reported to play a role in the pathogenesis and complications of cardiovascular disease through various different mechanisms, including inflammation [23, 34, 35]. Although we encountered no studies investigating the relationship between calprotectin and uric acid in hypertensive patients, the relationship between calprotectin and uric acid in the inflammatory process has been the subject of previous research. Several studies have reported that synovial-derived monosodium urate crystals cause an increase in calprotectin levels through neutrophil activation [36–38]. In addition, the S100 protein family and uric acid are part of a group of molecules known as “alarmin” and involved in tissue damage [39]. The current literature thus indicates the existence of a positive relationship between calprotectin and uric acid in the inflammatory process. The negative correlation observed in the present study may derive from the sensitivity to rapid proteolysis of the calprotectin heterotetramers.

Considering all these findings of the present study together, we think that the place of calprotectin in hypertension now deserves to be examined in a greater detail.

The limitations of this study include the low patient number and the fact that serum samples were used after being stored for some time and that different techniques used in calprotectin measurement could not be compared. In addition, evaluation of other endothelial markers together might have contributed to the interpretation of our findings.

In conclusion, the number of studies investigating calprotectin levels in individuals with newly diagnosed primary hypertension in which endothelial damage is not yet fully established is insufficient. The present study determined lower calprotectin levels in this patient group. Further comparative studies involving larger patient groups and patients at different stages of the hypertensive process, with and without complications, are now needed to clarify the place of calprotectin in the hypertension-associated inflammatory process. In addition, we think that the development of new methods with high clinical applicability in the measurement of calprotectin, or a review of current methods, especially for stored samples, will usefully contribute to clinical practice.

## Figures and Tables

**Table 1:** Baseline characteristics and laboratory findings of the study population.

Characteristics	Control (*n* = 38)	Hypertensive (*n* = 49)	*p*
Age (years)	44.66 ± 7.82	44.98 ± 11.12	0.880
Gender (female/male)	30/8	17/32	0.001
SBP (mmHg)	114.5 ± 9.4	155.0 ± 13.5	0.001
DBP (mmHg)	72.6 ± 8.0	96.6 ± 10.0	0.001
Glucose (mg/dL)	95.2 ± 7.3	96.7 ± 11.9	0.501
Creatinine (mg/dL)	0.65 ± 0.13	0.85 ± 0.17	0.001
eGFR (mL/min/1.73 m^2^)	108.9 ± 10.8	98.94 ± 15.74	0.04
Uric acid (mg/dL)	4.37 ± 0.92	5.74 ± 1.66	0.001
Sodium (mEq/L)	140.42 ± 1.97	140.9 ± 2.52	0.235
Potassium (mEq/L)	4.46 ± 0.33	4.46 ± 0.34	0.942
Albumin (g/dL)	4.56 ± 0.4	4.66 ± 0.4	0.104
Calcium (mg/dL)	9.4 ± 0.4	9.7 ± 0.4	0.030
CRP (mg/dL)	1.37 (0.3–9.4)	2.1 (0.5–23)	0.023
Calprotectin (ng/mL)	242.8 (72.4–524)	112.6 (67.4–389.7)	0.001

SBP: systolic blood pressure; DBP: diastolic blood pressure; eGFR: estimated glomerular filtration rate (was calculated using the Chronic Kidney Disease Epidemiology Collaboration); CRP: C-reactive protein. Continuous variables were reported as mean ± standard deviation or median (interquartile range), and categorical variables were reported as *n*. *p* value <0.05 was significant.

**Table 2:** Correlation between the serum calprotectin level and other findings of the study population.

Characteristics	All (87)		Control (*n* = 38)		Hypertensive (*n* = 49)	
	*r* ^*∗*^	*p*	*r* ^*∗*^	*p*	*r* ^*∗*^	*p*
Age (years)	0.161	0.136	0.143	0.393	0.241	0.096
SBP (mmHg)	−0.411	0.001	0.126	0.450	−0.096	0.512
DBP (mmHg)	−0.377	0.001	0.153	0.359	−0.01	0.945
Glucose (mg/dL)	−0.037	0.734	0.076	0.649	−0.140	0.336
Creatinine (mg/dL)	−0.204	0.059	0.104	0.536	0.026	0.860
eGFR (mL/dk/1.73 m^2^)	0.092	0.396	−0.057	0.733	−0.032	0.826
Uric acid (mg/dL)	−0.370	0.001	−0.342	0.038	−0.190	0.190
Sodium (mEq/L)	−0.131	0.226	−0.035	0.834	−0.145	0.321
Potassium (mEq/L)	0.141	0.194	0.124	0.459	0.276	0.055
Albumin (g/dL)	0.039	0.739	0.332	0.105	0.064	0.662
Calcium (mg/dL)	−0.293	0.009	0.059	0.750	−0.279	0.057
CRP (mg/dL)	−0.196	0.07	−0.123	0.470	−0.087	0.550

SBP: systolic blood pressure; DBP: diastolic blood pressure; eGFR: estimated glomerular filtration rate (as calculated using the Chronic Kidney Disease Epidemiology Collaboration); CRP: C-reactive protein. *p* value <0.05 was significant.

**Table 3:** Linear regression for serum calprotectin in the study population.

Covariates	Univariate					Multivariate				
	*t*	*p*	Beta	95% CI for *B*		*t*	*p*	Beta	95% CI for *B*	
				Lower	Upper				Lower	Upper
SBP	−4.57	<0.001	−0.44	−3.55	−1.40	−2.68	0.009	−0.54	−5.00	−0.74
DBP	−3.59	0.001	−0.36	−4.89	−1.40	1.17	0.245	0.24	−1.36	5.26
Creatinine	−2.15	0.035	−0.23	−312.11	−11.86	0.48	0.630	0.06	−120.7	198.04
Uric acid	−3.91	<0.001	−0.39	−50.28	−16.36	−2.30	0.024	−0.30	−42.43	−3.01
Calcium	−2.71	0.008	−0.30	−145.22	−22.24	−0.93	0.354	−0.11	−94.13	34.14

CI: confidence interval; SBP: systolic blood pressure; DBP: diastolic blood pressure. *p* value <0.05 was significant.

**Table 4:** Logistic regression for hypertension in the study population.

Covariates	Univariate			Multivariate		
	Beta	Exp(*B*) (95% CI)	*p*	Beta	Exp(*B*) (95% CI)	*p*
Gender^*∗*^	1.95	7.06 (2.66–18.75)	<0.001	1.15	3.14 (0.65–15.15)	0.154
eGFR	−0.56	0.95 (0.91–0.98)	0.003	−0.07	0.93 (0.89–0.98)	0.008
Calprotectin	−0.01	0.99 (0.99–1.00)	<0.001	−0.01	0.99 (0.98–1.00)	0.005
Calcium	2.00	7.42 (2.00–27.60)	0.003	0.97	2.63 (0.46–15.00)	0.276
Uric acid	0.86	2.37 (1.50–3.74)	<0.001	0.49	1.63 (0.88–3.03)	0.122

CI: confidence interval; eGFR: estimated glomerular filtration rate. *p* value <0.05 was significant.

## Data Availability

The “Google Drive” data used to support the findings of this study are available from the corresponding author upon request.

## References

[1] NCD Risk Factor Collaboration (NCD-RisC) (2017). Worldwide trends in blood pressure from 1975 to 2015: a pooled analysis of 1479 population-based measurement studies with 19·1 million participants. *The Lancet*.

[2] Khaddaj Mallat R., Mathew John C., Kendrick D. J., Braun A. P. (2017). The vascular endothelium: a regulator of arterial tone and interface for the immune system. *Critical Reviews in Clinical Laboratory Sciences*.

[3] Gkaliagkousi E., Douma S., Zamboulis C., Ferro A. (2009). Nitric oxide dysfunction in vascular endothelium and platelets: role in essential hypertension. *Journal of Hypertension*.

[4] Xiao L., Harrison D. G. (2020). Inflammation in hypertension. *Canadian Journal of Cardiology*.

[5] Tang E. H. C., Vanhoutte P. M. (2010). Endothelial dysfunction: a strategic target in the treatment of hypertension?. *Pfluegers Archiv European Journal of Physiology*.

[6] Montezano A. C., Dulak-Lis M., Tsiropoulou S., Harvey A., Briones A. M., Touyz R. M. (2015). Oxidative stress and human hypertension: vascular mechanisms, biomarkers, and novel therapies. *Canadian Journal of Cardiology*.

[7] Johne B., Fagerhol M. K., Lyberg T. (1997). Functional and clinical aspects of the myelomonocyte protein calprotectin. *Molecular Pathology*.

[8] Yang J., Anholts J., Kolbe U., Stegehuis-Kamp J., Claas F., Eikmans M. (2018). Calcium-binding proteins S100A8 and S100A9: investigation of their immune regulatory effect in myeloid cells. *International Journal of Molecular Sciences*.

[9] Miyamoto S., Ueda M., Ikemoto M. (2008). Increased serum levels and expression of S100A8/A9 complex in infiltrated neutrophils in atherosclerotic plaque of unstable angina. *Heart*.

[10] Li J., Huang L., Wang S., Zhang Z. (2018). Increased serum levels of high mobility group protein B1 and calprotectin in pre-eclampsia. *International Journal of Gynecology & Obstetrics*.

[11] Meuwis M.-A., Vernier-Massouille G., Grimaud J. C. (2013). Serum calprotectin as a biomarker for Crohn’s disease. *Journal of Crohn’s and Colitis*.

[12] Jarlborg M., Courvoisier D. S., Courvoisier D. S. (2020). Serum calprotectin: a promising biomarker in rheumatoid arthritis and axial spondyloarthritis. *Arthritis Research and Therapy*.

[13] Ma L.-P., Haugen E., Ikemoto M., Fujita M., Terasaki F., Fu M. (2012). S100A8/A9 complex as a new biomarker in prediction of mortality in elderly patients with severe heart failure. *International Journal of Cardiology*.

[14] Shabani F., Farasat A., Mahdavi M., Gheibi N. (2018). Calprotectin (S100A8/S100A9): a key protein between inflammation and cancer. *Inflammation Research*.

[15] Stríz I., Trebichavský I. (2004). Calprotectin - a pleiotropic molecule in acute and chronic inflammation. *Physiological Research*.

[16] Feng C., Tao Y., Shang T., Yu M. (2011). Calprotectin, RAGE and TNF-*α* in hypertensive disorders in pregnancy: expression and significance. *Archives of Gynecology and Obstetrics*.

[17] Trabulus S., Oruc M., Yavuzer S. (2020). Myeloid-related protein complex 8/14 increases in hypertensive patients with excessive renal damage. *Clinical Nephrology*.

[18] Kunutsor S. K., Flores-Guerrero J. L., Kieneker L. M. (2018). Plasma calprotectin and risk of cardiovascular disease: findings from the PREVEND prospective cohort study. *Atherosclerosis*.

[19] Williams B., Mancia G., Spiering W. (2018). 2018 practice guidelines for the management of arterial hypertension of the European society of cardiology and the European society of hypertension. *Blood Pressure*.

[20] Croce K., Gao H., Wang Y. (2009). Myeloid-related protein-8/14 is critical for the biological response to vascular injury. *Circulation*.

[21] McCormick M. M., Rahimi F., Bobryshev Y. V. (2005). S100A8 and S100A9 in human arterial wall. *Journal of Biological Chemistry*.

[22] Robinson M. J., Tessier P., Poulsom R., Hogg N. (2002). The S100 family heterodimer, MRP-8/14, binds with high affinity to heparin and heparan sulfate glycosaminoglycans on endothelial cells. *Journal of Biological Chemistry*.

[23] Jin M., Yang F., Yang I. (2012). Uric acid, hyperuricemia and vascular diseases. *Frontiers in Bioscience*.

[24] Wang Y., Hu J.-W., Lv Y.-B. (2017). The role of uric acid in hypertension of adolescents, prehypertension and salt sensitivity of blood pressure. *Medical Science Monitor*.

[25] Sabanayagam C., Shankar A. (2011). Serum calcium levels and hypertension among US adults. *Journal of Clinical Hypertension*.

[26] Sun H., Shi J., Wang H. (2013). Association of serum calcium and hypertension among adolescents aged 12-17 years in the rural area of Northeast China. *Biological Trace Element Research*.

[27] Hage F. G. (2014). C-reactive protein and hypertension. *Journal of Human Hypertension*.

[28] Kansui Y., Matsumura K., Morinaga Y. (2019). C‐reactive protein and incident hypertension in a worksite population of Japanese men. *Journal of Clinical Hypertension*.

[29] Wu Y., Li Y., Zhang C. (2014). S100a8/a9 released by CD11b + Gr1 + neutrophils activates cardiac fibroblasts to initiate angiotensin II-induced cardiac inflammation and injury. *Hypertension*.

[30] Leukert N., Vogl T., Strupat K., Reichelt R., Sorg C., Roth J. (2006). Calcium-dependent tetramer formation of S100A8 and S100A9 is essential for biological activity. *Journal of Molecular Biology*.

[31] Pröpper C., Huang X., Roth J., Sorg C., Nacken W. (1999). Analysis of the MRP8-MRP14 protein-protein interaction by the two-hybrid system suggests a prominent role of the C-terminal domain of S100 proteins in dimer formation. *Journal of Biological Chemistry*.

[32] Hoskin T. S., Crowther J. M., Cheung J. (2019). Oxidative cross-linking of calprotectin occurs in vivo, altering its structure and susceptibility to proteolysis. *Redox Biology*.

[33] Genre F., Rueda-Gotor J., Remuzgo-Martínez S. (2018). Association of circulating calprotectin with lipid profile in axial spondyloarthritis. *Scientific Reports*.

[34] Ndrepepa G. (2018). Uric acid and cardiovascular disease. *Clinica Chimica Acta*.

[35] Johnson R. J., Kang D.-H., Feig D. (2003). Is there a pathogenetic role for uric acid in hypertension and cardiovascular and renal disease?. *Hypertension*.

[36] Ryckman C., Gilbert C., de Médicis R., Lussier A., Vandal K., Tessier P. A. (2004). Monosodium urate monohydrate crystals induce the release of the proinflammatory protein S100A8/A9 from neutrophils. *Journal of Leukocyte Biology*.

[37] Ryckman C., McColl S. R., Vandal K. (2003). Role of S100A8 and S100A9 in neutrophil recruitment in response to monosodium urate monohydrate crystals in the air-pouch model of acute gouty arthritis. *Arthritis & Rheumatism*.

[38] Vedder D., Gerritsen M., Nurmohamed M. T., van Vollenhoven R. F., Lood C. (2020). A neutrophil signature is strongly associated with increased cardiovascular risk in gout. *Rheumatology*.

[39] Bianchi M. E. (2007). DAMPs, PAMPs and alarmins: all we need to know about danger. *Journal of Leukocyte Biology*.

